# Comparison between allergenic extracts of mites manufactured with raw materials from different sources

**DOI:** 10.1186/1939-4551-8-S1-A70

**Published:** 2015-04-08

**Authors:** Victor Espirito Santo Cunha, Tielli Magnus, Ruppert Ludwig Hahnstadt

**Affiliations:** 1 FDA Allergenic, Brazil

## Background

Allergenic extracts from different companies or even different batches of the same company can show great variation in allergens content and relative potency (RP). The objective of this study was to compare allergenic extracts of mites produced with raw material from different suppliers.

## Methods

Seven allergenic extracts produced with *Dermatophagoides pteronyssinus*from six suppliers were compared: extract A (USA), B and C (Spain), D (Argentina), E (Costa Rica), F and G (The Netherlands). This evaluation was carried out comparing the dosages of groups 1 and 2 major allergens of Dermatophagoides (ELISA - INDOOR Biotechnologies), RP (Competition ELISA - FDA Allergenic) and protein bands pattern (SDS-PAGE) among extracts.

## Results

The content of groups 1 and 2 allergens ranged from 21 (B) to 49 µg/mL (A), and from 4 (D) to 50 µg/mL (F), respectively. The RP varied from 0.25 (D) to 2.08 (F), when compare with the FDA Allergenic IHR. The protein bands pattern were characteristic of mite *D. pteronyssinus*, however, in F were observed some protein bands which had not seen in others extracts, and in G were observed only group 1 and 2 major allergens. The results showed that there are significant differences between extracts made with raw material from different suppliers and even between extracts produced with raw material from the same supplier (extracts F and G). The results found in this study cannot be explained by differences in the manufacturing process, since all extracts were produced by the same process. The extracts with the greatest quantities of major allergens and relative potency (A and F) were produced with semi-purified raw material (both with more than 99% of purity), while the other extracts were produced with mite whole culture (mites, feces and culture media).

## Conclusions

It was concluded that the quality or purity of raw material used in the manufacturing process of allergenic extracts directly influences the final product quality, and therefore, analytical methods to control the relative potency, in order to assure batch to batch reproducibility, are essential to effectiveness and safety of allergenic products.

